# Genetic improvement of Pacific white shrimp [*Penaeus (Litopenaeus) vannamei*]: perspectives for genomic selection

**DOI:** 10.3389/fgene.2015.00093

**Published:** 2015-03-24

**Authors:** Héctor Castillo-Juárez, Gabriel R. Campos-Montes, Alejandra Caballero-Zamora, Hugo H. Montaldo

**Affiliations:** ^1^Departamento de Producción Agrícola y Animal, División de Ciencias Biológicas y de la Salud, Unidad Xochimilco, Universidad Autónoma MetropolitanaMexico City, Mexico; ^2^Departamento El Hombre y su Ambiente, Unidad XochimilcoUniversidad Autónoma MetropolitanaMexico City, Mexico; ^3^Departamento de Genética y Bioestadística, Facultad de Medicina Veterinaria y Zootecnia, Universidad Nacional Autónoma de MéxicoMexico City, Mexico

**Keywords:** *P. vannamei*, growth, survival rate, disease resistance, genomic selection

## Abstract

The uses of breeding programs for the Pacific white shrimp [*Penaeus (Litopenaeus) vannamei*] based on mixed linear models with pedigreed data are described. The application of these classic breeding methods yielded continuous progress of great value to increase the profitability of the shrimp industry in several countries. Recent advances in such areas as genomics in shrimp will allow for the development of new breeding programs in the near future that will increase genetic progress. In particular, these novel techniques may help increase disease resistance to specific emerging diseases, which is today a very important component of shrimp breeding programs. Thanks to increased selection accuracy, simulated genetic advance using genomic selection for survival to a disease challenge was up to 2.6 times that of phenotypic sib selection.

## Introduction

Shrimp production is an important activity both in economic terms and from the perspective of its contribution to human nutrition. Total world shrimp production had a value of approximately $13.6 billion USD in 2013. The most important shrimp production regions in the world are located in Asia, principally China, India, Vietnam, Indonesia, and Bangladesh, and in the Americas, primarily in Ecuador, Brazil, and Mexico (Food and Agriculture Organization of the United Nations [FAO], 2014).

Genetic improvement is an important option for increasing profitability in agriculture and aquaculture (Gjedrem et al., 2012). Several shrimp breeding programs have been implemented in a number of countries, some of which have been reviewed by Neira (2010) and Rye (2012).

The increasing importance of disease to shrimp farming worldwide has stimulated research for developing breeding programs for increasing disease resistance/tolerance to disease. Evidence for successful selection for disease resistance exists in shrimp for Taura virus and other diseases (Cock et al., 2009; Lightner et al., 2012). In a few cases, alternative programs which combine mass selection in communally raised animals with recovery of family identification using DNA markers have been used in order to perform mating that avoids excessive increases in inbreeding rates and to select animals in the presence of disease, allowing natural selection to act toward increased genetic resistance (Rocha, 2012). Other programs have used experimental challenges combined with mass selection in successive generations and obtained increases in the genetic resistance to White Spot Syndrome Virus (Cuéllar-Anjel et al., 2012).

## Shrimp Breeding Programs

Although many commercial shrimp breeding programs are not fully described, most are based on population structures typical for aquaculture breeding, using full- and half-sib family structures (Gjedrem et al., 2012). Most shrimp breeding programs focus on the improvement of growth traits and general survival rate. Some have concentrated on selection for specific diseases. These traits can be improved genetically by within-line selection.

The Maricultura del Pacífico hatchery commercial line population was started in 1998 in Mexico, from a heterogeneous population formed by a mix of domesticated lines and wild shrimp from several origins. The main efforts have been directed toward instrumenting a breeding program to develop a genetic line oriented towards improving profitability of biomass production under the production conditions predominating in the northwest part of Mexico. Since biomass production depends on harvesting body weight and survival rates, these two traits have been incorporated as the broodstock selection criteria. The relative importance of each of these traits in the selection index used is 5:1 for harvesting body weight and survival, respectively, which is based on economic studies under the main production system conditions found in Mexico.

Each year from 2003 to 2010, an average of 15,445 shrimp obtained from 130 females and 93 males were evaluated for body weight at harvesting. These evaluations were performed in 2–4 ponds where commercial like production conditions are reproduced. Starting in 2009, survival from 65 to 130 days of age also has been genetically evaluated. Mating is designed based on the use of shrimp from the selected families with the higher selection index values. Hence, broodstock animals come on average, from the upper 27% families in the case of males, and from the upper 53% families in the case of females. In addition to these selection procedures, within-family selection is performed at various growing stages based on approximations to the individual body weight. This is carried out in the genetic nucleus, under strict biosecurity conditions. Animals coming from the within-family selection procedures are the animals ultimately used as the next generation broodstock. Reproduction techniques are based on artificial insemination only, using two females per male. These procedures have been described by Castillo-Juárez et al. (2007) and by Campos-Montes et al. (2013), and some methodological implications have been discussed by Montaldo et al. (2013). Considering the time needed to evaluate animals and to obtain mature broodstock ready to use, the complete production cycle required to yield a new generation (generational interval) is 1 year.

## Genetic Progress

Genetic progress has been evaluated in several shrimp selection programs. Hetzel et al. (2000) estimated the selection response per generation for 6-month weight in *Peneaus japonicus* at 8.3%. Andriantahina et al. (2012) estimated the genetic response per generation for 5-month weight in *Litopenaeus vannamei* at 10.7%. The genetic response for body weight at harvesting (130 days of age) in the Maricultura del Pacífico commercial line has been evaluated using linear models (BLUP-animal model). The estimated genetic gain as a linear trend from 2003 to 2010 represents an increment of 18.4% of the average body weight for the period. For survival, the estimated genetic gain as a linear trend from 2004 to 2010 was also positive (1.56%).

## Potential of Genomic Selection for Disease Resistance

Diseases are major constraints for aquaculture production. As vaccination is not an option in shrimp and management contention measures are frequently unfeasible, genetic selection is considered a possible option in fighting many diseases in *P. vanname*i and other shrimp species. Genomic selection (GS) increases accuracy if compared to conventional selection, by taking advantage of both between and within-family variance, in situations where family testing for disease is used in sibs of the actual selection candidates, in order to avoid the introduction of the pathogen into the breeding nucleus population (Villanueva et al., 2011).

We used SelAction software (Rutten et al., 2002) and methods developed by Dekkers (2007) to deterministically simulate selection response in *P. vannamei* using dense genetic marker arrays (chips). This was done to assess the potential effects of GS in a breeding program oriented to the improvement of disease resistance. This simulation considered the context of a typical shrimp breeding program based on (sib) family selection. We used seven heritability values: 0.01, 0.05, 0.10, 0.20, 0.30, 0.40, and 0.50 for survival under experimental infestation of shrimp produced in breeding shrimp populations of different size, and relatively high selection intensities, if compared to that from our actual breeding program (Campos-Montes et al., 2013). This allows us to evaluate the possible genetic response for survival to different pathogens, which may include a wide range of viral as well as bacterial infections. The considered proportion of common full-sib environmental effects was 0.15 for all cases.

Accuracy from GS was obtained with the formula developed by Daetwyler et al. (2010), assuming a genome size of 28 Morgans, and an effective population size of 50. This yielded a value for the number of independent chromosome segments close to 324, which may be considered conservative (Villanueva et al., 2011); therefore accuracy from GS was probably not overestimated. The proportion of the additive genetic variance explained by markers to reflect the preliminary stage of development of SNP chips in shrimp was also conservatively set at a value of 0.64 which is below the value of 0.80 used for the 50 K SNP chip for cattle (Daetwyler, 2009).

All breeding populations were derived from 30 males and 38 females, with an incomplete nested structure, similar to that described by Montaldo et al. (2013), to produce 150 families to be tested. Population sizes corresponding to 6, 50, and 100 shrimp per female were 900, 7,500, and 15,000 measured offspring, respectively.

Three breeding strategies were considered here for between family selection: (1) phenotypic information, (2) GS (based on using contemporary training and testing population of similar size to avoid an increase of generation interval), and (3) combined selection program which uses both genomic and phenotypic information.

**FIGURE 1:**
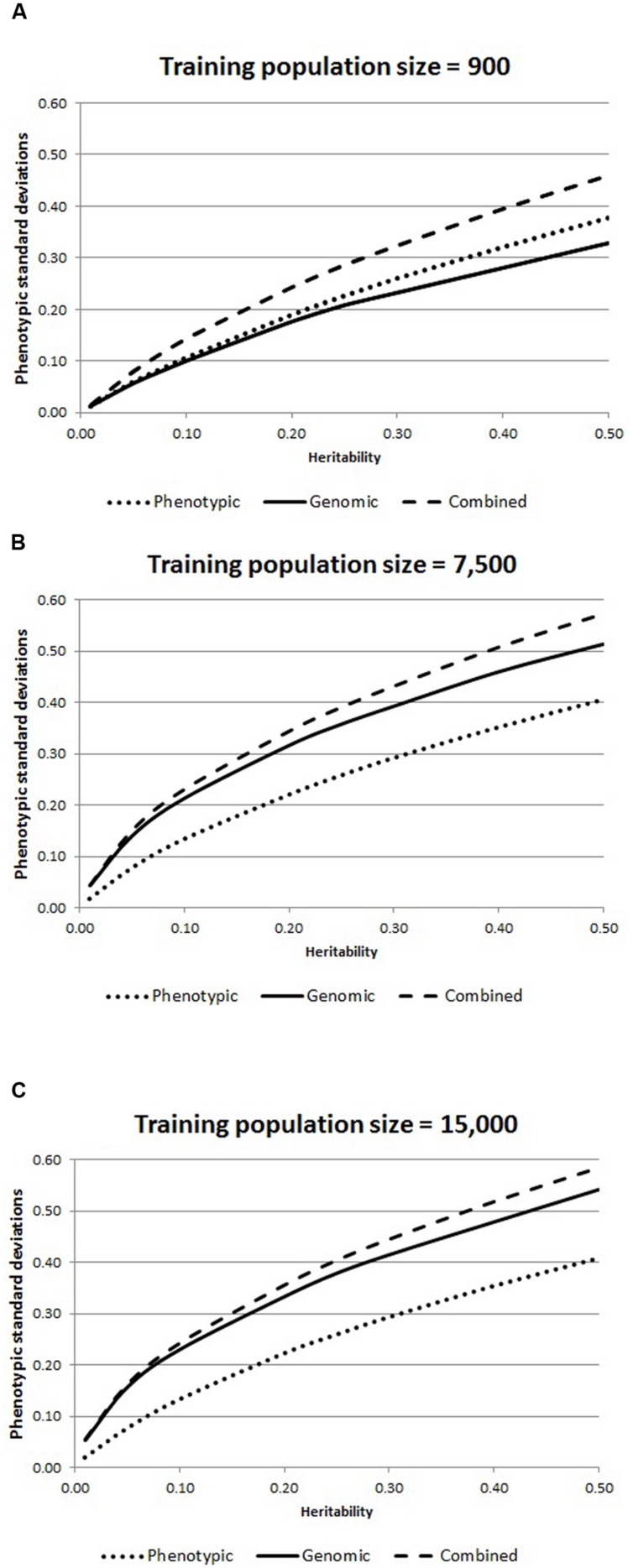
**Expected responses (phenotypic SD) to phenotypic, genomic and combined selection programs according to heritability value for different training population sizes (A, B and C) in *Penaeus vannamei***.

Results (**Figure 1**) show that the potential of GS to develop lines with improved disease resistance is high. GS and combined selection programs had large increases in selection responses measured in phenotypic standard deviation units for survival, in populations of size 7,500 and 15,000, but less for the population of size 900. Ratio from combined and GS programs, with respect to phenotypic programs, increased with lower heritability values. These ratios for populations of size 7,500 were 2.6, 1.7, 1.6, 1.5, 1.4, and 1.4 for combined selection for heritability values of 0.01, 0.10, 0.20, 0.30, 0.40, and 0.50, respectively, and only slightly lower for GS. Analogous ratios for combined and GS relative to phenotypic selection for a population of size 15,000 were very similar. Results for combined selection for a population of size 900 showed an advantage over phenotypic selection with lower ratios (from 1.2 to 1.3), while ratios for GS were slightly below 1. These results indicate that with a training population of adequate size (probably of less than 7,500), GS could more than double the rate of selection response for survival to specific diseases, which is higher than previous estimates for GS programs in the context of aquaculture breeding for continuous traits (Nielsen et al., 2011). Interestingly, the relative advantage was greater for smaller heritability values. This is promising for improving survival to disease challenges in aquaculture species, which often have low heritability values (Yáñez and Martínez, 2010).

In this preliminary GS shrimp evaluation, we compared all the programs at the same selection intensity. This may give some unrealistic detriment to GS programs, because GS would allow selection of individual animals at higher selection intensities, when the individual identification of candidates is possible. Therefore, not all the potential advantages of GS and larger candidate populations to increase selection response were included in this study. Combined and GS responses were similar for populations of sizes 7,500 or 15,000, indicating that GS is capturing almost all the variation explained by the phenotypes, making GS more accurate. A more detailed comparison may include changes in inbreeding rates, and the optimization of the different factors affecting selection response. Advances in shrimp genomics (Yu et al., 2014; Zhang et al., 2014) may lead to the future development of SNP chips for *P. vannamei*, making the possibility of performing GS in this species more viable in the near future.

Within the framework of our preliminary calculations, GS for survival rates to disease challenges in shrimp may lead to large increases in selection responses across a wide range of heritability values.

## Conclusion

The use of the so-called “classic” or conventional methods of quantitative genetics to genetically improve the Pacific white shrimp has allowed for continuous progress of great value to increase the profitability of the shrimp industry in several countries and other aquaculture species. Recent advances in such areas as genomics will allow, in the near future, for the development of animal breeding methods, which may increase, and hence accelerate shrimp genetic progress. In particular, these novel techniques may help increase disease resistance to specific emerging diseases, which is today a very important aspect in shrimp breeding programs.

## Conflict of Interest Statement

The authors declare that the research was conducted in the absence of any commercial or financial relationships that could be construed as a potential conflict of interest.
